# Doing challenging research studies in a patient-centred way: a qualitative study to inform a randomised controlled trial in the paediatric emergency care setting

**DOI:** 10.1136/bmjopen-2014-005045

**Published:** 2014-05-15

**Authors:** Kerry Woolfall, Bridget Young, Lucy Frith, Richard Appleton, Anand Iyer, Shrouk Messahel, Helen Hickey, Carrol Gamble

**Affiliations:** 1 Department of Psychological Sciences, University of Liverpool, Liverpool, UK; 2 Department of Health Service Research, University of Liverpool, Liverpool, UK; 3 The Roald Dahl EEG Unit, Paediatric Neurosciences Foundation, Alder Hey Children's NHS Foundation Trust, Liverpool, UK; 4 Department of Paediatric Emergency Medicine, Alder Hey Children's NHS Foundation Trust, Liverpool, UK; 5 Department of Biostatistics, University of Liverpool, Liverpool, UK

**Keywords:** Accident & Emergency Medicine, Qualitative Research, Medical Ethics

## Abstract

**Objective:**

To inform the design of a randomised controlled trial (called EcLiPSE) to improve the treatment of children with convulsive status epilepticus (CSE). EcLiPSE requires the use of a controversial deferred consent process.

**Design:**

Qualitative interview and focus group study.

**Setting:**

8 UK support groups for parents of children who have chronic or acute health conditions and experience of paediatric emergency care.

**Participants:**

17 parents, of whom 11 participated in telephone interviews (10 mothers, 1 father) and 6 in a focus group (5 mothers, 1 father). 6 parents (35%) were bereaved and 7 (41%) had children who had experienced seizures, including CSE.

**Results:**

Most parents had not heard of deferred consent, yet they supported its use to enable the progress of emergency care research providing a child's safety was not compromised by the research. Parents were reassured by tailored explanation, which focused their attention on aspects of EcLiPSE that addressed their priorities and concerns. These aspects included the safety of the interventions under investigation and how both EcLiPSE interventions are used in routine clinical practice. Parents made recommendations about the appropriate timing of a recruitment discussion, the need to individualise approaches to recruiting bereaved parents and the use of clear written information.

**Conclusions:**

Our study provided information to help ensure that a challenging trial was patient centred in its design. We will use our findings to help EcLiPSE practitioners to: discuss potentially threatening trial safety information with parents, use open-ended questions and prompts to identify their priorities and concerns and clarify related aspects of written trial information to assist understanding and decision-making.

**Strengths and limitations of this study**

- This is the first study to provide detailed insight into how parents perceive deferred consent in the challenging paediatric emergency care setting. Practitioners can use the findings to assist parental understanding and decision-making by discussing potentially threatening trial safety information with parents and using open-ended questions and prompts to identify and discuss their priorities and concerns.
- Our interview and focus group study involved parents of children with a range of acute and chronic health conditions who had experience of the emergency care setting. The findings are therefore potentially transferable to other trials that propose a deferred consent approach in paediatric emergency care.
- Our findings demonstrate the value of using qualitative methods at the pre-trial stage to make clinical trials more patient centred and to provide evidence to help challenge assumptions about approaches to consent that might otherwise go unchallenged.
- The proposed trial (called EcLiPSE) was hypothetical and not all parents in our sample had children who had experienced the particular condition that is the focus of the trial. Our sample is also likely to comprise parents with an interest in research, which may not reflect the target EcLiPSE sample.
- Children were not involved in our study. Research is required to explore their perceptions of deferred consent in emergency care trials.

## Background

The design of research studies often requires a balance to be struck between what is ethically and pragmatically acceptable and what is scientifically ideal.^1–3^ Qualitative research has a potential role to inform this balance, particularly in challenging settings where some trials might otherwise be regarded as being too problematic to conduct. For example, in paediatric settings, there are relatively few clinical trials to inform the development of emergency care interventions to save the lives of children.^4^ Paediatric accident and emergency care trials are fraught with ethical and practical difficulties.^5^ Freely given informed consent of a patient before any research procedures are implemented is a key principle of good clinical practice to protect patient rights, safety and well-being.^6^ ^7^ The process of informed consent requires an exchange of information with ‘ample time and opportunity to inquire about details of the trial and decide whether or not to participate in the trial’.^8^ This information exchange is often impossible in the emergency care setting, where seeking prospective consent would delay the administration of time-critical interventions. Moreover, the delays needed to fulfil requirements for informed consent may reduce the effect of any interventions.^9^ As children (<16 years) cannot legally provide consent for a trial of investigational medicinal products, informed consent in this setting refers to the ‘proxy’ consent that is sought from parents or legal guardians. Even when interventions in the emergency setting are not so time-critical, there are ethical concerns about the quality of parental informed consent, as their capacity to understand trial information is likely to be compromised by the stressful situation.^4^ ^10^ In 2008, UK legislation was amended to address such issues by enabling consent to be deferred in children's clinical trials^11^ when the following conditions are met: (1) treatment is required urgently; (2) urgent action is required for the purposes of the trial; (3) it is not reasonably practicable to obtain consent prospectively and (4) an ethics committee has given approval to the procedure under which the action is taken. Consent for the child's participation in the trial can therefore be sought from parents or legal guardians *after* his/her enrolment and the administration of trial interventions. In this situation, consent is being sought for the child to continue in the trial and for his/her data to be retained and included in the analyses.^11^ ^12^

Despite legislation enabling deferred consent, its use remains controversial. Patients in such trials do not have an opportunity to veto the investigational interventions because these will have already been performed by the time deferred consent is sought.^13^ ^14^ Internationally, there is a lack of research that describes public attitude towards deferred consent^13^ and how to make it appropriate to the needs of parents, children and practitioners. A trial conducted almost a decade ago compared the effectiveness of buccal midazolam versus rectal diazepam for the emergency treatment of status epileptics in children.^15^ As it was not deemed appropriate to seek consent from a parent while their child was in a tonic–clonic seizure, consent was deferred until as soon as practically possible after treatment. Consultation took place as part of the trial to explore the acceptability of deferred consent with participating families, although the findings of this consultation were never reported. Researchers in the USA using research consent waivers^16^ are required to use a community consultation approach whereby the researchers are required to consult with representatives of the community from which participants are derived, as well as post public notices of the study protocol, risks, benefits and results.^16^ ^17^ However, the Food and Drug Administration (FDA) has recently issued guidance on conducting emergency research without consent^18^ in response to varied practice, including wide variations in the consultation methods used.^19^ ^20^

Qualitative research may provide a more systematic approach to consultation for emergency care trials; such research can also facilitate exploration of public and patient opinion to inform approaches to consent in the emergency setting. Studies have shown how qualitative research can inform trial development in challenging settings, including the identification of barriers and potential solutions to successful recruitment^21–23^ and acceptability of approaches to consent procedures.^24^ ^25^ Historically, there has been a paucity of such research, despite its potential to help trialists understand the complexities and challenges arising from the social contexts in which trials are based.^26^ A recent systematic mapping review of qualitative research in the clinical trials setting indicated^27^ that while such work had considerable potential to inform trials, this potential is often lost because the qualitative study findings are too late to inform the partner trials. The reviewers argued that initiating qualitative research at the design stage of partner trials would help to increase the impact of this type of work, thus benefiting trials and, ultimately, patients.^28^

We identified the need for a clinical trial to improve the treatment of children suffering from convulsive status epilepticus (CSE). CSE is the most common and serious neurological emergency in children.^29^ ^30^ Although there is a very low risk that children treated for CSE will subsequently die (<1%),^31^ these children are at increased risk of irreversible morbidity, including chronic drug-resistant epilepsy and neurodisability related not only to the condition and its cause but also its management.^32^ The current management of CSE depends on a national algorithm wherein two doses of a benzodiazepine medication are administered initially.^33^ If the seizures continue, a second and longer acting anticonvulsant is used. For many years this has been phenytoin, but there is no randomised controlled evidence to support its use. There are several serious adverse effects associated with phenytoin use including hypotension, cardiac arrhythmias (which may prove fatal), hepatotoxicity, phlebitis, severe tissue extravasation injury (the ‘purple glove syndrome’) and Steven Johnson's syndrome.^34^ Intravenous levetiracetam has shown potential to be a safe and effective alternative to phenytoin.^35^ ^36^ Recent evidence has suggested that it not only terminates CSE but can also be injected quicker, it has milder, more transient side effects and does not have the cardiac or hepatic toxicity seen with phenytoin.^37^ We therefore designed EcLiPSE (Emergency use of Levetiracetam vs Phenytoin in Status Epilepticus), an unblinded pragmatic multicentre randomised trial to compare two treatments (intravenous levetiracetam and intravenous phenytoin) for the termination of acute, prolonged tonic–clonic seizures, including CSE in children aged between 6 months and 18 years.

Challenges in conducting the trial were identified by practitioners and patient public involvement (PPI) representative within the trial team during the design stage and included: a vulnerable target population (children aged between 6 months and 18 years); the need for the intervention to be delivered during a medical emergency; insufficient time to obtain informed consent prior to the intervention and levetiracetam not being the standard second-line anticonvulsant used to treat status epilepticus. In addition, previous trials conducted since the introduction of legislation enabling deferred consent in paediatric trials^11^ have involved comparisons of investigational interventions in current standard use. EcLiPSE is breaking new ground in using deferred consent within a trial that compares an established treatment, with a treatment that is not yet in standard use.^25^ We reasoned that qualitative research could help us identify how best to approach these challenges in a way that was family-centred and ethically acceptable. We designed our qualitative study to explore the views of parents on EcLiPSE, our approach to seeking deferred consent in the emergency care setting and the content of the patient information sheet (PIS), with the aim of using the findings to inform our deliberations on EcLiPSE's design and associated grant and research ethics committee applications.

## Methods

We used a focus group and semistructured interviews with parents of children with acute and chronic conditions who had experience of their children being admitted to a paediatric accident and emergency department for urgent medical care. This work was conducted as part of a wider study (called CONNECT) investigating consent methods in paediatric and neonatal emergency care trials.

The CONNECT advisory group and EcLiPSE trial development team developed and reviewed an interview topic guide and draft EcLiPSE PIS. The topic guide covered key areas indicated within a review of the literature and previous CONNECT study findings^25^ and the EcLiPSE trial team identified further topics pertinent to this trial. Topics included: approaches to consent in the emergency care setting; parental understanding and decision-making; length and content of information provided in the PIS; trial design and acceptability of deferred consent. We created a separate section of questions for bereaved parents to explore their views and recommendations on whether and how parents should be approached about a clinical trial after a child's death.

Children eligible to participate in EcLiPSE either have chronic epilepsy and may be susceptible to CSE, or may present with a first prolonged tonic–clonic seizure. The team agreed that it was important to ensure participation of families who had experienced treatment of this medical emergency as well as those without such experience. We contacted a range of UK parent support groups for parents of children with acute and chronic conditions to request their help in identifying suitable parents for our qualitative study. In addition, support groups for bereaved parents and conditions associated with CSE in children (eg, Dravet syndrome, Lennox-Gastaut syndrome) were purposively sampled to ensure that the views of such parents were included.^38^ Identified gatekeepers (eg, support group research coordinators) were asked to send CONNECT invitations to their members via email, or place the request on their website or Facebook page. The inclusion criteria stated that parents should have experience of paediatric emergency care. Parents who registered an interest via email were sent a CONNECT information sheet, consent form and a copy of the EcLiPSE PIS. To acknowledge childcare responsibilities and personal preference, we provided parents with the option to take part in a telephone interview or focus group. Parents were asked to indicate whether they were bereaved in order to tailor the interview questions appropriately. We only asked bereaved parents questions about approaches to consent in EcLiPSE in the situation that a child had died; we felt that it would be difficult for non-bereaved parents to understand the complexities of this situation and make appropriate recommendations to inform trial protocol. All interviews were semistructured using a topic guide with open-ended questions and unstructured prompts to facilitate free-flowing conversation and explore unanticipated topics. Discussion was participant centred to ensure that the content reflected their own priorities and views on EcLiPSE rather than the researchers’.

KW conducted all interviews (including the focus group) and led the analysis. The focus group and subsequent interview sessions were digitally audiorecorded, transcribed and anonymised. Respondent validation was used whereby previously unanticipated topics raised by participants were added to the topic guide and discussed with additional participants as interviewing and analysis progressed.^39^ For example, changes to the PIS suggested by parents during the initial focus group were presented by KW during subsequent interviews for discussion and review.^40^ ^41^ To assist this process, KW reviewed early transcripts and the developing coding framework and discussed these in meetings with members of the CONNECT advisory group (LF and BY) and the EcLiPSE development team (RA, CG, HH, SM and AI).^42^ Recruitment stopped when new data ceased adding to the analysis, indicating that data saturation was achieved.^43^ KW contacted parents who were not interviewed (due to data saturation), explaining why their participation in an interview was no longer required, thanking them for their interest in the study and requesting their involvement in future related research. Analysis was broadly interpretive and iterative, referring back and forth between the developing analysis and new data for evidence of parents’ views on approaches to recruitment and consent in EcLiPSE.^40^ ^41^ Themes were therefore inductively derived from the data. While the analysis was informed by the constant comparison approach of grounded theory, the focus was modified to fit with the criterion of catalytic validity, whereby findings should be relevant to future research and practice.^41^ ^44^ KW read interview transcripts several times to compare within and between transcripts.^40^ ^41^ We used QSR NVivo V.10 software to assist in the organisation and indexing of coding and transcripts.

## Results

Of the 63 parent support groups contacted by telephone, 14 (22%) agreed to participate and sent the study invitation to parents by email or placed the request on their website or Facebook page. Gatekeepers at 8 (13%) support groups declined to participate as they did not feel the study was appropriate for their members. For example, their group supported parents of children who may have died before arriving at an accident and emergency department. The remaining 41 (65%) groups did not respond to telephone messages.

Twenty-five parents registered interest in an interview. Data saturation^42^ was reached at the point where 17 parents had been interviewed by telephone (11 parents: 10 mothers, 1 father) or focus group (6 parents: 5 mothers, 1 father). The 17 parents were recruited across eight UK support groups for parents of children with acute and chronic conditions including: meningitis, autism, congenital diaphragmatic hernia (CHD), bronchomalacia, quadriplegia, acquired brain injury, epilepsy and Dravet syndrome. Six parents (35%) were bereaved and seven (41%) had children who had experienced a tonic–clonic seizure. Six (35%) parents had experienced being approached about their child taking part in a paediatric or neonatal clinical trial (four provided consent, one declined and one child was ineligible). The remaining six support groups who agreed to participate did not result in any uptake from their members.

### Acceptability of deferred consent

Early in the interviews and focus group and prior to any discussion of EcLiPSE, KW read a general definition of deferred consent to parents:

Due to the need to treat a patient in an emergency without delay, or parents not always being present when a child needs treatment, it is not always appropriate or possible to obtain consent before a child is entered into a trial. Instead UK legislation allows consent to be sought as soon as possible afterwards. This is for permission to use the data already collected and to continue in the trial. This is called deferred consent. Deferred consent is a relatively new approach to seeking consent in the UK.

KW then prompted parents to explore their prior knowledge and views on this method of consent. Two parents had heard of deferred consent, but neither of them had personal experience of it. Although the majority (n=15) were unfamiliar with this approach to consent, they responded positively to the description. Parents described how deferred consent was a sensible solution to seeking consent in the emergency care setting: it “makes sense really, doesn't it?” (P8, telephone interview, not bereaved). In this context, parents emphasised the need for research for the common good, often describing how they supported the approach in the emergency setting to inform the development of treatments for children in the future.

It’s the right direction to go, really, because quite often, um, you just don’t have the time or the situation, and the data is valuable (P17, mother, telephone interview, bereaved)

If it helps other children then that's brilliant you know (P9, mother, telephone interview, bereaved)

Without that data, you know, you don't move forward (P 2, mother focus group, not bereaved)

Many parents trusted practitioners to do the best for their child and viewed research-related decisions as part of the practitioners’ role in an emergency situation when parents’ capacity to understand what was being proposed would be limited as a result of the intense anxiety about their child's situation:

But then in that situation you are kind of a bit fuzzy anyway, and you think well they know what they are doing, so you know we sort of trust them to do their job (P 9, mother, telephone interview, bereaved)

That [deferred consent] to me is fine because it's er obviously based on a decision taken by doctors who are the best people to ask, if you like, under that situation and, and that's a decision that they make so that's, that's absolutely fine, yeah, I wouldn't have a problem with that (P11, father, telephone interview, bereaved)

Although the majority of parents felt that deferred consent was broadly acceptable, two parents anticipated that they would be initially shocked or “uneasy” (P2, focus group, not bereaved) if they were informed that their child had been entered into a trial without their prior consent. Views on the acceptability of deferred consent were dependent on the nature of the trial and the level of perceived risk parents attributed to the intervention being administered. As the following focus group excerpt illustrates, deferred consent for observation studies was viewed as more acceptable than for a drug trial, particularly if the drug was unknown to the parent or involved drug administration by injection.

P1 (mother, not bereaved): It depends on what exactly they're doing, whether they're just taking a blood sample or whether they're injecting them with something that I don't know, um I don't know what it is even

P5 (father, bereaved): Whether it's drugs or whether it's just sort of an easy test

P2 (mother, not bereaved): Obs (observational study, which does not involve any intervention)

P1 (mother, not bereaved): How much risk do you want to take?

### Responses to the ECLIPSE trial

Following the general discussion of deferred consent, KW shifted the focus to EcLiPSE. She asked each participant if they had read the EcLiPSE PIS. She then read out key excerpts from this document including the trial aims, a description of drugs involved, safety profile and rationale for the use of deferred consent, before prompting parents for their responses to these aspects of the trial. One parent described administering a drug to a child without prior parental consent as “ethically very difficult” (P12, mother, telephone interview, not bereaved). Most parents (n=9) expressed reservations about EcLiPSE due to the safety profile of phenytoin as described on the information sheet. As the following focus group excerpt illustrates, these parents were initially shocked at hearing about the side effects of phenytoin and discussed how these stood out from other information provided on the sheet and had caused alarm:

P4 (mother, not bereaved): I'd be, I'd be scared if they’ve written, ‘Very serious unpleasant side effects’ part

P5 (father, bereaved): *Yeah*

P6 (mother, not bereaved): It really hits you, doesn’t it, that?

P4 (mother, not bereaved): *It does, yeah*

However, parents’ opinions about the trial and its use of deferred consent appeared to change after KW explained how phenytoin (which is the drug associated with the serious side effects that parents had expressed concern about) is currently used in clinical practice and that outside of EcLiPSE this drug would be the standard treatment for prolonged seizures. Although the point that phenytoin was in routine use had already been available to parents on the PIS, it was not until KW verbally reiterated this information, and explained that the aim of the trial was to see whether or not levetiracetam is a more effective alternative phenytoin, that parents’ initial concerns appeared to subside.

Facilitator: What would your initial thoughts be about this trial?

P 14 (mother, not bereaved): *I think I'd be a bit scared...*

Facilitator: …So your child would have received phenytoin routinely if the seizures had not stopped. This can cause very unpleasant and serious side-effects. Studies of levetiracetam in adult emergency situations suggest that it may be an alternative rescue medicine to phenytoin. There have been no major side effects reported with the use of levetiracetam. So that's sort of why they're doing the trial. It's to see if this drug, which some hospitals are using, might be better than phenytoin, which everybody's using and they know can have nasty side effects. Would that help if they explained that to you a bit more?

P 14 (mother, not bereaved):Yeah, so from that point of view, that sounds a lot better... That, that would be the pretty much perfect explanation to make a mum turn around and go, it's so they're doing everything they can to make sure my child is safe and to try and stop any side-effects

After KW explained that phenytoin was the standard treatment for prolonged seizures, all parents stated that they would have provided deferred consent for EcLiPSE. Parents cited their strong belief in the need for research to advance children's emergency medicine as informing their position: “we're not gonna advance unless we try” (P12, mother, telephone interview, not bereaved). Some parents who had expressed initial concerns about the description of drug's side effects on the PIS went on to indicate that they wanted a ‘truthful’ description of potential drug side effects: “I'd rather just hear the truth” (P5, father, focus group, bereaved). Parents therefore emphasised the importance of open explanation and discussion when broaching the trial, in addition to the written PIS. They described the content and quality of verbal information and explanation as key in helping parents to understand the aims and risks of the trial: “How it's actually explained to parents at that point will have a huge impact” (P10, mother, telephone interview, not bereaved).

Parents pointed to how the outcome of a child's status epilepticus was likely to be a key factor in how future parents might respond to EcLiPSE when approached about it and their willingness to provide deferred consent. Some (n=4) suggested that if a child does not recover or experiences serious side effects, parents could react angrily and feel their voluntariness has been compromised by the use of deferred consent:

It depends, if your child is the one that has the very serious side effects or your child is the one that it worked for (P3, mother, focus group, not bereaved)

I suppose your sticky wicket here is if it's helping and if it's not, isn't it? (P7, mother, telephone interview, bereaved parent)

It wouldn’t be my response...because there is nothing that I can do, I mean I can withdraw consent all I like, but it might make me very cross (P17, mother, telephone interview, bereaved)

### When to approach parents for deferred consent

When asked for their views on the ‘best time’ for practitioners to approach parents for deferred consent in an emergency situation*,* parents suggested that this should be done “sooner rather than later” (P1, mother, focus group, not bereaved). However, they also recommended that practitioners should (where possible) wait until the child was stable before approaching parents: “obviously when things are stable to approach the parents because you're in a period of calm then” (P5, telephone interview, bereaved). Parents suggested that practitioners should gauge when is appropriate for each family on a case-by-case basis. They recommended that practitioners should consult with someone close to the family, such as the bedside nurse to help establish the appropriate timing of the initial invitation.

It probably would help if you’ve got someone who’s been quite close with the, with the family, to sort of help to gauge whether or not it’s an appropriate time (P8, mother, telephone interview, not bereaved)

I do genuinely feel that in 99 per cent of the cases, if it was… if you approached them in the right way and at the right time then there wouldn't be a problem (P5, father, focus group, bereaved)

### Approaching bereaved parents

KW explained to bereaved parents why it was necessary for the trial team to approach bereaved parents in EcLiPSE:

Children who receive emergency care are often very poorly and sadly some will not survive. Sometimes a child has been entered into a trial before they had passed away and the doctor or nurse would then come and talk to the parents to see how they feel about consenting for their child's data to be included in the trial. The reason they ask bereaved parents for deferred consent is because without including all children, trial findings won't provide a full picture of how safe or effective a drug is. The findings will be biased. Doctors and nurses want to understand what it is like for parents in this situation and whether they should approach them about the trial

KW then asked bereaved parents for their views on approaching parents for consent for a child's data to be included in EcLiPSE after the child had died. All but one of these six parents indicated that parents should be approached for deferred consent. The parent who expressed reservations described how approaching bereaved parents in this situation would add to their grief: “my child's gone, yeah, I'm grieving. I don't want you to send me a letter and remind me of something you were doing when I was in that bad place” (P7, mother, telephone interview, bereaved). The remaining parents explained how they would wish to be provided with the opportunity for their child's data to be used in the study. Many described how they strongly supported medical research to inform research for the common good and to help prevent other parents from experiencing a child's death.

If it helps another child in the future, then all the better you know...it happened so why not use the, whatever information you gained from it to help somebody else. And I think the people that I know that are bereaved would probably feel pretty much the same (P9, mother, telephone interview, bereaved)

You basically want to do everything you can to stop it from happening to anyone else really (P17, mother, telephone interview, bereaved)

Bereaved parents described the individuality of grief and how this posed difficulties in making broad recommendations that would be appropriate for all bereaved parents. However, many (n=5/6) explained that approaching parents for consent after a child's death, whether it be conducted by letter, telephone or in person, should not be too soon after death “they have to be um not just straight in there, er they would leave… would have to leave it at the time” (P11, father, telephone interview, bereaved). Parents recommended that a doctor or nurse known to the family should broach the subject and emphasised the considerable care and sensitivity that an approach to parents in such circumstances would demand. Again, parents emphasised how practitioners should individually gauge each situation to establish when it is appropriate to approach each individual family. As the following quotes illustrate, parents acknowledged that practitioners approaching parents in this situation should be aware that parent's responses may be unpredictable due to the grief they would be suffering:

You have to understand that you're dealing with a completely irrational time, and there’s no, nothing really makes sense and nothing is logical… so I think it has to be approached with care, but I mean, I, I certainly wouldn’t mind it (P 17, mother, telephone interview, bereaved)

I don't even know if there is a right way, because even, you know, how someone would talk to me, and how someone would talk to my husband, we would both react completely different (P9, mother, telephone interview, bereaved)

Some you wouldn’t, you wouldn’t approach at all, but I think you have to leave that to the discretion of the nurse” (P11, father, telephone interview, bereaved)

Five bereaved parents described how practitioners seeking deferred consent should be prepared to address potential concerns from parents that the interventions administered as part of the trial may have contributed to their child's death:

It might have contributed to making them even more poorly than they actually were, obviously you wouldn't, you wouldn't be very happy about that (P5, father, focus group, bereaved).

### Terminology and written information

Parents spoke of how the EcLiPSE PIS (see appendix A, web only file) was generally clear: “I don’t think there’s anything that’s particularly confusing on there” (P8, telephone interview, not bereaved) and the correct length for the emergency care setting: “You don't want a really big sheet to have to sit and read through when your child's not very well anyway so I think what's in it is enough for, for what you'd need to know” (P13, mother, telephone interview, not bereaved). However, they also pointed to particular medical terms in the PIS, which they felt would be a potential barrier to parent–practitioner communication and parental understanding of trial information. Parents recommended simplifying some of the medical language and viewed this as important to help parents understand the trial information when faced with it in a stressful emergency care situation. However, at the same time, parents indicated that there was a linguistic balance to be struck and that it was important to avoid language that might be perceived as patronising.

It's worded in a way that might go over people's heads, particularly if they're in a distressed state… Not a dummy's guide ’cause that, that's just really insulting but basically make it a lot, lot simpler and not so medicalised (P12, mother, telephone interview, not bereaved)

Parents of children with epilepsy who were familiar with the trial drugs recommended the use of the brand name Keppra rather than the generic name levetiracetam, as this was the name they used and would recognise if presented with the PIS: “I can never pronounce that so I call it the brand name, which is Keppra” (P9, mother, individual interview, bereaved). Changing from generic drug names to brand names was also recommended by several other participants whose children did not have seizures, as they also found the generic names difficult to pronounce: “I hate it when doctors call drugs by their full name, I want to hear the brand name because that's what I know and that's what I can say” (P10, mother, telephone interview, not bereaved). Parents also suggested changes to sentence structure in the PIS and requested an improved explanation to clarify that both medicinal products had previously been used to effectively treat children: “there probably needs to be more of a paragraph about how both of the drugs that are in the trial erm have been used erm successfully” (P4, mother, focus group, not bereaved) and the need for legal information on what parents should do if they have a complaint: “you could have legal action” (P6, mother, focus group, not bereaved). We provide original and revised (when interviews were complete) versions of the PIS (see appendices A and B, web only files) to illustrate how the findings of the qualitative study informed the development of this document.

## Discussion

We believe EcLiPSE is the first UK trial comparing investigational medicinal products to propose a deferred consent approach since this approach was legislated in 2008.^11^ Our findings provide insight into the views of parents experienced in this setting. The majority of parents in our sample were unfamiliar with deferred consent, yet responded positively to a general description of the method. When discussing deferred consent generally, parents questioned their capacity to provide an informed consent decision when their child was ill.^4^ ^45^ They described how they trusted practitioners to make research-related decisions on their behalf and viewed deferred consent as an appropriate way to seek consent in emergency situations and thereby enable the future development of interventions to treat critically ill children.^46^ In this context, parents indicated that study and intervention type, safety information and route of administration impacted on their views on the acceptability of the consent method. From a parent's perspective, these factors could all be seen as markers of risk related to their child's participation in the trial. Indeed, parents viewed observational studies as safer than trials of medicinal products and therefore a more acceptable study type to use deferred consent. For a few parents, trials that involved unfamiliar drugs also raised concerns about child safety. These findings helped to inform the design of EcLiPSE, the PIS and the consent-seeking process.

When the focus of the discussion moved to the specifics of EcLiPSE and the use of deferred consent in this trial, parents questioned the acceptability of deferred consent in this context and many expressed initial shock and concern about the safety of the trial linked to the description of drug risks provided on the PIS. However, when KW read and discussed sections of the information sheet related to parents’ priority for safety and anxieties about risk (eg, information related to safety and that both drugs were used as part of routine clinical practice), parents appeared to be reassured. After this tailored explanation, most parents indicated that they would be willing to provide deferred consent for their child's participation in EcLiPSE, as they wished to contribute to advances in medical research. There are several potential explanations for this marked switch in parents’ views. Although parents stated that they had read the one page PIS before the interview, they may not have fully read or understood it,^47^ ^48^ so they were unclear that both drugs had previously been used effectively to stop tonic–clonic seizures. The content of the PIS may have been insufficient. For example, the use of the word ‘routinely’ may be inadequate to convey how the drugs had previously been used in clinical practice. However, it was arguably the interviewer's explanation of how phenytoin was the standard treatment for prolonged seizures that appeared to focus parents’ attention on aspects of the trial which addressed their priorities and concerns,^49^ namely a child's safety. Our findings highlight how trial practitioners need to discuss^50^ potentially threatening information with parents to identify their priorities^49^ and clarify related aspects of written trial information to assist understanding and decision-making. As parents may struggle to voice their concerns in recruitment discussions with practitioners,^51^ these qualitative findings will be used to inform EcLiPSE recruiter training. Training will focus on helping practitioners to identify and respond to parents’ priorities,^50^ ^52^ and include the use of open-ended questions and prompts.^21^ ^49^

In line with our previous findings from the wider CONNECT study, which investigated practitioners’ views on deferred consent in this setting,^25^ the timing of the recruitment discussion may impact on parental responses to the method of consent. Parents, particularly those who had been bereaved, emphasised the need for practitioners to gauge when it is appropriate to discuss the trial. Consultation with the clinical team may help practitioners establish appropriate timing for a trial discussion. Our findings add to the existing literature, which suggests that bereaved parents do wish to be informed about a trial in the aftermath of their child's death,^53^ while serving as a reminder that a minority of parents feel such disclosure could add to their grief. Although we would emphasise that it is very unlikely that children treated in EcLiPSE will die in status epilepticus (<1% mortality rate),^31^ our findings will inform approaches to consent with this vulnerable group of parents. Our findings draw further attention to the need for care in gauging when to explain to bereaved parents that some of their child's treatment had been administered as part of a trial and to seek their consent for the child's data to be used in the analyses. Parents cautioned that some time should be allowed to elapse following a child's death and that the approach should be conducted by a nurse or doctor known to the family. Importantly, our findings also indicate the highly variable and unpredictable nature of grief following the death of a child and how practitioners need to be allowed to use their judgement to accommodate the needs of individual parents. The EcLiPSE protocol will be developed to facilitate practitioners in assessing each family individually and to initially obtain information on how the family is coping from colleagues and bereavement counsellors before making a decision about whether or not and when to contact a family. However, further research is required to explore potential conflicts of interest or privacy issues when practitioners seek information from colleagues about the coping of bereaved families. Our findings suggest that decisions to approach for consent should be balanced against the potential burden that a recruitment discussion may pose to parents who are already emotionally and psychologically distressed, and the likelihood that it will be very difficult for practitioners to ascertain if and when it is appropriate to approach such vulnerable families when seeking deferred consent for all families, EcLiPSE practitioners should explore parents’ views of the trial and be prepared to respond to parents who are concerned that participation may have been a contributing factor in their child's death or poor recovery. These findings are relevant to other paediatric and neonatal clinical trials in this setting. As it is unlikely that children treated in EcLiPSE will die, excluding bereaved parents without obtained deferred consent is unlikely to impact on trial findings. However, it is important to acknowledge that the exclusion of this group of children may jeopardise study results^54^ for emergency care trials that experience higher rates of mortality.

To assist understanding and parent–practitioner communication, parents emphasised the need for simple and clear information, without oversimplification. This linguistic balance may be difficult for trial teams to achieve without input from parents or patients.^55^ We amended the language used in the PIS, removing the repetition of medical terminology and using brand names rather than generic names for medicinal products (appendix B, web only file). We also removed the word ‘routinely’ from the PIS and used our findings to improve the written explanation that both trial drugs were commonly used in clinical practice. Parents indicated that a one page PIS was of sufficient length and that they would not wish to read much more than this when their child was ill. These findings confirm those from other trials that have indicated that the PIS should be short, and add to these findings by providing insights on what parents regard as user-friendly language.^56^ Parents also approved the open and comprehensive description of drug risks in the PIS. Therefore, the description of drug risks for phenytoin was not changed in the redrafted information sheet (see appendices A and B, web only file).

### Strengths, limitations and implications

Our findings provide insights to help practitioners when seeking deferred consent in the paediatric emergency care setting. As with many qualitative studies, our sample was relatively small; however, data saturation was reached,^42^ and we involved parents of children with a range of acute and chronic health conditions who had experience of the emergency care setting. Our findings may therefore be transferable to other trials that propose a deferred consent approach in paediatric emergency care. Little is known about what practitioners should do in the event that a child dies before deferred consent is sought.^57^ These findings contribute to this important and under-researched area and demonstrate the value of using qualitative methods in helping to make challenging clinical trials more family or patient centred.^21^ ^58^

As we wanted to use the findings to inform the design of a future trial, inevitably the trial was hypothetical at this stage. Not all parents in our sample had children who had experienced the particular condition (CSE) that is the focus of EcLiPSE. To enable the successful but sensitive recruitment of bereaved parents, we did not restrict inclusion to parents of children who had died of CSE, or restrict eligibility by time since death. Nevertheless, our sampling of such parents was designed to reflect the variation in the experience of parents whose children are likely to participate in EcLiPSE. Our sample is likely to comprise parents with an interest in research who may be more easily reassured than the wider population of parents. This interest in research may not reflect the potential EcLiPSE sample. As part of the wider CONNECT study, we have found that views on deferred consent differed depending on whether or not the practitioners were experienced in this consent method.^25^ Those who were not experienced held negative preconceptions of deferred consent, whereas those who had experience of the method were receptive to the method, describing how deferred consent had improved recruitment, parental decision-making and parent–practitioner relationships in this challenging setting. Further research will be conducted with parents who are actually approached for deferred consent when EcLiPSE recruitment begins, to explore whether their responses differ from the views of parents in this sample. This work will aim to include parents of children who die before consent for EcLiPSE is sought. Research embedded within trials will also help to explore whether parental responses to recruitment vary depending on how well their child recovers. Findings from research to explore the views of parents approached for deferred consent when EcLiPSE recruitment begins will be incorporated into trial information and practitioner training as part of an iterative process^40^ ^41^ to inform trial recruitment and approaches to consent in this challenging trial.

Children were not involved in our study. Research is required to explore their views on the use and appropriateness of deferred consent in emergency care trials. Involving children experienced in deferred assent may be challenging, as there are few UK trials which have used this method,^25^ and assent may not have been sought if a child was recovering or still sedated at the point of recruitment discussion with parents. Moreover, it is highly unlikely that children will have knowledge of trial participation if parents have not informed them.^59^

Most UK funding bodies, including the National Institute for Health Research (NIHR), require PPI with the aim of advancing research, including its design, conduct and dissemination.^60^ PPI is a prerequisite for funding; it refers to patients and the public working as research partners or contributing advice on whether and how research is designed and conducted. Currently, PPI often involves a small number of PPI representatives, who have been selected in a variety of ways and whose experience may not be relevant to a particular trial, acting as coapplicants and steering group members and contributing to decisions about the trial design. For EcLiPSE, we felt that a qualitative study was necessary in addition to PPI to ensure that the trial was informed by systematic exploration and analysis of the perspectives of a diverse group of parents, whose experiences were pertinent to the trial. This provided insight into how parents may view EcLiPSE when they are approached about it and helped us to identify strategies to enhance recruitment and parent understanding.^28^ ^61^ We anticipate that this insight could not have been achieved through the involvement of PPI representatives alone. However, qualitative research requires funding and sufficient time for the development of research protocols, ethical review procedures, as well as to recruit participants, and to collect, analyse and interpret the data. Funding opportunities for this type of research are limited. UK funding bodies should consider how best to resource qualitative research to inform the design of challenging trials at the pre-trial stage to ensure that trials are feasible and more patient or family centred.
